# Influences of Fermented Corn Straw Fiber on Performance and Nutrient Utilization in Different Breeds of Finishing Pigs

**DOI:** 10.3390/ani14233393

**Published:** 2024-11-25

**Authors:** Rui Han, Feng Yong, Xin Fang, Chun Zhang, Haitian Yang, Dongsheng Che, Hailong Jiang

**Affiliations:** 1 College of Animal Science and Technology, Jilin Agricultural University, Changchun 130118, China; hanrui0409@163.com (R.H.); yongfeng0485@163.com (F.Y.); xinfangkiligs@163.com (X.F.);; 2 Ministry of Education Laboratory of Animal Production and Security, Changchun 130118, China; 3 Jilin Provincial Key Laboratory of Animal Nutrition and Feed Science, Changchun 130118, China; 4 Jilin Provincial Swine Industry Technical Innovation Center, Changchun 130118, China; 5 Assets and Equipment Department, Changchun Sci-Tech University, Changchun 130600, China

**Keywords:** breed, dietary fiber, finishing pig, nutrient, growth performance

## Abstract

This study aimed to explore the effects of dietary fiber level and breed on the growth performance, nutrient utilization, intestinal morphology, slaughter performance, and meat quality of finishing pigs using fermented corn straw (FCS) as the fiber source. The experiment employed a 2 × 4 factorial design, selecting 96 Songliao Black (SLB) and Duroc × Landrace × Yorkshire (DLY) crossbred finishing pigs (a total of 192 pigs, with an initial body weight of 60.52 ± 4.59 kg) randomly assigned by breed to four dietary treatment groups (A: 2.92% crude fiber; B: 4.82% crude fiber; C: 6.86% crude fiber; D: 9.01% crude fiber). The results showed that DLY finishing pigs had higher final weight (FW), average daily gain (ADG), and average daily feed intake (ADFI) in both finishing stages 1 and 2 compared to SLB pigs (*p* < 0.05), while the ratio of feed to weight gain (F/G) showed no significant differences (*p* > 0.05). Compared to the basal diet, increasing the dietary fiber level to 4.82% improved FW and ADG in both SLB and DLY finishing pigs (*p* < 0.05) and reduced F/G (*p* < 0.05). Additionally, SLB finishing pigs had lower ether extract (EE) digestibility but higher crude fiber (CF) and acid detergent fiber (ADF) digestibility than DLY (*p* < 0.05). Dietary fiber level and breed exhibited an interaction effect on dry matter (DM) and crude protein (CP) digestibility in finishing pigs (*p* < 0.05). At a dietary fiber level of 4.82%, villus height, crypt depth in the jejunum, and cecal volatile fatty acid (VFA) concentrations were increased in both SLB and DLY finishing pigs (*p* < 0.05). Dietary fiber level and breed showed an interaction effect on cecal VFA production in finishing pigs (finishing stage 1; *p* < 0.05). The dietary fiber level of 4.82% increased loin eye area (LA) (*p* < 0.05) and decreased backfat thickness (BT) (*p* < 0.05) in both SLB and DLY finishing pigs. Dietary fiber level and breed had an interaction effect on LA in finishing pigs (*p* < 0.05). SLB pigs had higher muscle redness (a*), shear force, and contents of crude protein (CP), EE, saturated fatty acid (SFA), and polyunsaturated fatty acids (PUFA) than DLY (*p* < 0.05). Increasing the dietary fiber level improved pH_24h_ and reduced drip loss and shear force in both SLB and DLY finishing pigs (*p* < 0.05). Dietary fiber level and breed showed an interaction effect on pig meat color and drip loss (*p* < 0.05). In conclusion, FCS is a beneficial source of dietary fiber for SLB and DLY pigs. Its proper addition can enhance the growth performance, carcass traits, and meat quality in fattening pigs.

## Simple Summary

The utilization of fibrous agricultural by-products not only improves the intestinal development and physiological function of monogastric animals but also increases their added value as agricultural by-products. This study used fermented corn straw as a fiber source to partially replace the diet in varying proportions and compared its effects on the production performance and nutrient utilization of local breed pigs and commercial pigs. The objective is to provide reasonable suggestions for the different dietary fiber requirements of pigs with different genetic backgrounds and to establish a theoretical basis for the resource utilization of agricultural by-products.

## 1. Introduction

The rational utilization of fibrous agricultural by-products (of plant origin) in the pig industry is crucial for enhancing production efficiency and optimizing dietary composition in animal husbandry. In recent years, with the shift in feeding strategies towards sustainable nutrition, various unconventional feedstuffs containing high dietary fiber, such as sugar beet pulp, wheat bran, soybean hulls, and corn husk, have been increasingly used in pig diets [1,2,3]. Corn straw, the by-product remaining after corn harvest (with an annual production in China exceeding 379 million tons), is partially used in ruminant production, while approximately two-thirds is still treated as waste and incinerated [4]. Due to their nutritional characteristics (high fiber; low protein and energy), the feeding value of corn straw for pigs is very restricted [5]. Therefore, the utilization of corn straw and other straw feed resources in monogastric animals, especially in pig production, has been an ancient problem and remains a current pressing concern. Recent research has demonstrated that microbial fermentation treatment can degrade some indigestible fiber components in corn straw, increase certain soluble monosaccharides and polysaccharides, as well as small peptides and enzymes, thereby improving its nutritional characteristics and enhancing its feeding effect in animal production [6,7,8]. Although pigs lack the ability to secrete fiber-degrading enzymes in their digestive tract, their large intestines host numerous microorganisms with cellulolytic activity, similar to the rumen of ruminants [9,10]. These microorganisms can partially or completely ferment certain fiber components to produce volatile fatty acids for energy supply [10]. Previous research has shown that pig diets must contain a certain amount of fiber to support the normal physiological function and development of the digestive tract [11]. Our previous studies also found that an appropriate amount of fermented corn straw (FCS; 10% of the diet) can improve litter performance, milk protein, and lactose content of sows without negatively affecting the utilization of nutrients [12,13].

The Songliao black (SLB) pig is an excellent native pig breed in northern China. Compared to Duroc × Landrace × Yorkshire (DLY) pigs, SLB pigs demonstrate high resistance to rough feeding, strong adaptability, and high reproductive performance.

Considering that pigs of different breeds and physiological stages may have specific requirements for fiber nutrition, this study compared the effects of replacing the diet with commercial FCS at different ratios on the production performance and nutrient utilization of local breed pigs (SLB) and commercial pigs (DLY). The aim is to provide reasonable suggestions for the different dietary fiber requirements of pigs with different genetic backgrounds, and to establish a theoretical basis for the resource utilization of agricultural by-products, promoting efficient feeding of finishing pigs of different breeds.

## 2. Materials and Methods

The Laboratory Animal Welfare and Ethics Committee of Jilin Agricultural University (Changchun, China) approved all protocols (Approval Number: 20220405003) used in this experiment.

### 2.1. Dietary Fiber

FCS was utilized as the experimental dietary fiber source, provided by Jilin Jiabailian Biotechnology Co., Ltd., Changchun, China. The harvested corn straw underwent dusting, cleaning, and crushing in accordance with the production process, followed by the addition of polypeptide to eliminate mold. *Lactobacillus plantarum* and *Lactobacillus buchneri* (purchased from Laihui Biotechnology Co., Ltd., Suzhou, China) were added to seal fermentation for 72 h. The nutritional composition of FCS is shown in Table 1.

### 2.2. Experimental Diets

In this study, the basal diet was replaced by FCS in different proportions (6%, 12%, and 18%). The composition of the experimental diets for two breeds of fattening pigs can be found in Table 2.

### 2.3. Animals and Experimental Design

After a 5 d adaptation period, 192 healthy and castrated male pigs (96 pigs per breed) with an initial body weight of 60.52 ± 4.59 kg were selected from two breeds (SLB and DLY) and randomly divided into 4 experimental groups (3 replicates per group and 8 pigs per replicate) according to different pig breeds.

SLB pigs are grouped as SA, SB, SC, and SD, while the DLY pigs are grouped as DA, DB, DC, and DD. Group SA and DA represent the control group, and pigs in this group were fed the basal diet with a crude fiber (CF) content of 2.92%. Group SB and DB, group SC and DC, and group SD and DD were the trial groups; pigs in these groups were fed with 6%, 12%, and 18% FCS (with CF contents of 4.82%, 6.86%, and 9.01%, respectively). The experiment was divided into two fattening stages: stage 1 (60–90 kg) and stage 2 (90–120 kg), each lasting for a period of 40 days, resulting in a total experimental duration of 80 days. At the end of the experiment, the growth performance, nutrient digestibility, serum biochemical, intestinal morphology, slaughter performance, and meat quality were determined for both fattening stages.

Pigs in each group were kept in houses with an ambient temperature of 20 ± 2 °C and humidity of 40–60%. Pigs were fed three times a day (06:00, 11:00, and 16:00) to prevent abnormal hunger until the next feeding and could drink freely. Individual animals were weighed on the first and last day of each fattening period to assess their growth performance. Additionally, average daily feed intake (ADFI) and health status of each pig were recorded.

### 2.4. Sample Collection

Fecal samples were collected 7 days before the end of each fattening stage. Immediately after collection, feces from each pen were placed into individual valve bags, and a 10% H_2_SO_4_ solution was evenly added to fix feces. Blood samples were taken from the precaval vein one day before the end of each fattening stage. Then, the blood samples were centrifuged at 3500× *g* at 4 °C for 15 min. Following additional centrifugation, the resulting serums were stored at −80 °C until tested. At the end of each fattening stage, four pigs from each replicate were selected and slaughtered following a 12 h fasting period (48 pigs of SLB and DLY were slaughtered at each stage). The small intestine segments were promptly isolated, rinsed with saline, and then fixed in 4% paraformaldehyde fix solution for subsequent morphological analysis. Cecal digesta samples were collected and stored in cryotube at −80 °C for the analysis of volatile fatty acids (VFAs). The longissimus thoracis (LT) of the left carcass was utilized for assessing meat quality, nutrient composition, and fatty acid.

### 2.5. Chemical Analysis

All samples underwent duplicate chemical analysis, with any discrepancies exceeding 5% prompting repeated testing. The dry matter (DM), crude protein (CP), ether extract (EE), and ash contents were determined for feed, fecal, and LT samples using the methods of Association of Official Analytical Chemists (AOAC, 2006) [15]. The numbers of these protocols are as follows: DM (AOAC official method 930.15), CP (AOAC official method 954.01), EE (AOAC official method 920.39), and ash (AOAC official method 924.05). The CF (AOAC official method 978.10) in the feed and fecal was also determined using the AOAC (2006) method. Acid detergent fiber (ADF) and neutral detergent fiber (NDF) in the feed and fecal were determined using a fiber analyzer, according to a previous report [16].

### 2.6. Biochemical Marker Assays in Serum

The serum biochemical parameters, including glucose (GLU), triglyceride (TG), total cholesterol (TC), glutamic oxalacetic transaminase (AST), glutamic-pyruvic transaminase (ALT), albumin (ALB), total protein (TP), blood urea nitrogen (BUN), high-density lipoprotein (HDL), and low-density lipoprotein (LDL) were analyzed using commercially available biochemical kits (Nanjing Jiancheng Biotechnology Co., Ltd., Nanjing, China). The analysis was performed on the automatic biochemical analyzer (BS-400, Mindray, Shenzhen, China) according to the manufacturer’s instructions. The detailed information of these kits is as follows: GLU (Cat. No. F006-1-1), TG (Cat. No. A110-1-1), TC (Cat. No. A111-1-1), AST (Cat. No. C010-1-1), ALT (Cat. No. C009-3-1), ALB (Cat. No. A028-1-1), TP (Cat. No. A045-3-2), BUN (Cat. No. C013-2-1), HDL (Cat. No. A112-1-1), and LDL (Cat. No. A113-1-1).

### 2.7. Intestinal Physiology and Volatile Fatty Acid

The fixed samples of the duodenal, jejunal, and ileal tissues were trimmed, dehydrated by graded anhydrous ethanol, and subsequently embedded in paraffin blocks. Cross-sections of each sample were cut to an approximate thickness of 5 μm and stained with hematoxylin and eosin (H&E). Histological sections were examined with an Olympus BX51 microscope coupled with an Integrated Digital Imaging Analysis System (Olympus Co., Tokyo, Japan). Nine crypt depths and villus heights were measured, and the average values were calculated. Following this, the ratio of villus height to crypt depth (V/C) was calculated.

The VFA analysis was carried out as previously described [17]. The samples were thawed on ice, and approximately 1 g of thawed sample was mixed with 8 mL of deionized water. This mixture was then thoroughly homogenized by vortexing for 1 min and centrifuged at 10,000× *g* and 4 °C for 15 min. A volume of 70 μL from the mixture was analyzed for VFAs using a GC-System (Agilent Technologies 7890A-G3440A-GC System; Agilent Technologies, Santa Clara, CA, USA). The temperature of the injector port and detector port was set at 220 °C and 230 °C, respectively.

### 2.8. Carcass Traits and Meat Quality

After slaughter, the hot carcass weights were recorded and used to calculate dressing percentage (DP). The left side of the carcass was utilized for measuring the average skin thickness, average backfat thickness (BT, the shoulder, lumbar, and last rib), and loin-eye area (10th to 11th ribs, length × width × 0.7).

The color attributes including lightness (L*), redness (a*), and yellowness (b*) were measured on the cross-section (3 locations) of the LT (the last rib) for each pig (24h post-slaughter), using a MiniScan EZ (Model: 4500L, 45°/0° illumination system, Hunter Lab Corp., New York, NY, USA) calibrated against a standard white plate (measurement aperture: 31.8 mm, illuminant: D65, standard observer angle: 10°), with color development time after cutting being 10–15 min. After calibrating with pH 4.6 and 7.0 buffers, a pH meter (pH-STAR, SFK-Technology, Herlev, Denmark) was used to measure the pH value of LT muscle samples from the left side of the carcass at the last rib at 24 h postmortem.

For drip loss determination, LT samples (about 25 g) were trimmed to 2 cm × 3 cm × 5 cm along the fiber direction, weighed (initial weight) after removal of fat and surface moisture, then suspended in inflated plastic bags for 24 h at 4 °C, and reweighed (final weight, FW). Drip loss percentage = (initial weight − final weight)/initial weight × 100% [13].

To measure the cooking loss, the muscle sample was vacuum packaged and cooked for 30 min in a water bath at 80 °C until the internal temperature reached 70 °C. After cooling at room temperature (20 °C) for 30 min, the surface moisture was removed with paper, and then the cooked sample was weighed. Cooking loss was determined by calculating the percentage change in weight.

The shear force was determined for the LT sample at the second last rib using the previously established procedure. The samples were placed in plastic bags and cooked in a 75 °C water bath until they reached an internal temperature of 70 °C, after which they were cooled to room temperature. Using a cylindrical core borer, 3 slices (1.27 cm diameter) were cut from each sample, and these slices were sheared perpendicularly to the fiber orientation by a texture analyzer (TA.XT.Plus, Godalming Stable Microsystems, Surrey, UK) equipped with a thin Warner–Bratzler blade with a V-shaped slot. The blade had a thickness of 3.0 mm ± 0.2 mm, a test speed of 2.00 mm/s, and a trigger force of 0.0050 kg [18].

Fatty acid composition was determined according to the previously reported method [19]. For fatty acid extraction, LT samples weighing 50 mg were combined with a methanol/water solution (volume ratio of 2:5, 280 μL) and methyl tertiary butylether (400 μL). Prior to extraction, a 6 mm grinding bead was introduced into each sample. The samples were then homogenized at −10 °C using a tissue crusher at 50 Hz for 6 min, followed by ultrasonic treatment in an ice-water bath for 30 min at 40 kHz and 5 °C, and subsequently placed at −20 °C for 30 min. After centrifugation, 350 μL of the supernatant was collected, dried under nitrogen, resuspended in a 1:1 isopropanol/acetonitrile solution (100 μL), and subjected to sonication (5 °C, 40 kHz, 5 min) on ice to extract lipids. The supernatants were then collected for ultra-high performance liquid chromatography-tandem mass spectrometry (UHPLC-MS/MS) analysis after spinning at 13,000× *g* at 4 °C for 15 min. The analysis was performed using a Thermo UHPLC-Q Exactive HF-X system equipped with an Accucore C30 column (100 mm × 2.1 mm i.d., 2.6 μm) from Waters, Milford, MA, USA. A binary solvent system was used, comprising mobile phase A (50% water and 50% acetonitrile solution containing 10 mmol/L ammonium acetate) and mobile phase B (10% acetonitrile, 2% water and 88% isopropanol solution containing 2 mmol/L ammonium acetate). The mobile phase gradient was as follows: 0–4.0 min, 60% B; 4.0–12.0 min, 85% B; 12.0–15.0 min, 100% B; 15.0–17.0 min, 100% B; 17.0–18.0 min, 35% B; and 18.0–20.0 min, 35% B. The column temperature was maintained at 40 °C, and the sample injection volume was 2 µL.

### 2.9. Statistical Analysis

Data were analyzed using the Yijk = µ + αi + βj + αi × βj + ɛijk model, where µ is the population mean, αi is the effect of pig breed (i = 1, 2), βj is the crude fiber level (j = 1, 2, 3, 4), αi × βj is the linear interaction between pig breed and the crude fiber level, and ɛijk is the residual effect. The data were analyzed using SPSS version 27.0 (IBM Corp., Armonk, NY, USA). All data were checked for normal distribution and homogeneity of variance using Levene’s test. The main effects (pig breed and crude fiber level) and their linear interaction effects were analyzed using two-way ANOVA. Differences between the 8 treatments were analyzed by multiple comparisons using Tukey’s method. Differences at a *p* < 0.01 were considered highly significant, at a *p* < 0.05 were considered significant, and at a 0.05 ≤ *p* < 0.1 were considered a trend. The results were expressed as the mean and standard error of the mean.

## 3. Results

### 3.1. Effects of Fiber Level on Growth Performance in Different Breeds of Finishing Pigs

The effects of dietary fiber level (fermented corn straw instead of basal diet) on the growth performance of finishing pigs with different breeds are shown in Table 3. The results showed that the growth performance of finishing pigs was influenced by different breeds. The SLB pigs had lower FW, average daily gain (ADG), and ADFI than the DLY pigs in two stages of fattening (*p* < 0.05). The feed-to-gain (F/G) ratio was slightly lower in the SLB pigs compared to the DLY pigs during both fattening stages. With the increase in fiber level, FW and ADG were initially increased and then decreased in two stages, and group B was significantly higher than groups C and D (*p* < 0.05), as well as higher than the control group. The high fiber level increased the ADFI and F/G. Different breeds and fiber levels had a significant impact on the ADG and ADFI of finishing pigs at two stages (*p* < 0.05). The FW and ADG (fattening stages 1 and 2) in the SC and SD groups did not show significant differences compared to those in the SA group, whereas the FW and ADG (fattening stages 1 and 2) in the DD groups were significantly lower than those in the DA and DB groups. The interaction between different breeds and dietary fiber levels only had a significant effect on ADG (*p* < 0.05).

### 3.2. Effects of Fiber Level on Nutrient Digestibility in Different Breeds of Finishing Pigs

The effects of fiber level on nutrient digestibility in different breeds of finishing pigs are listed in Table 4. The study on the effects of different breeds on the nutrient digestibility of finishing pigs revealed that the EE digestibility of SLB pigs was significantly lower than that of DLY pigs (*p* < 0.05), while significantly higher CF, ADF, and NDF digestibility than DLY pigs (*p* < 0.05) during fattening stage 1. Furthermore, the CF and ADF digestibility in SLB pigs were significantly higher than those in DLY pigs in stage 2 (*p* < 0.05). The digestibility of DM, CF, ADF, and NDF in groups C and D was significantly lower than those in groups A and B (*p* < 0.05). Additionally, the digestibility of CF in group B was significantly lower than that in group A (*p* < 0.05). In fattening stage 2, the CP digestibility of group B was significantly higher than that of group A (*p* < 0.05), and also the EE digestibility was higher than that of group A. The comparison results of eight treatment groups showed that the fiber level and breed had significant effects on the digestibility of EE, CF, and ADF in both stages (*p* < 0.05). Dietary fiber level and breed had an interactive effect on DM and CP digestibility in fattening stages 1 and 2 (*p* < 0.05).

### 3.3. Effects of Fiber Level on Serum Biochemical Parameters in Different Breeds of Finishing Pigs

As indicated in Table 5, serum biochemical parameters were influenced by fiber level and breed. The serum LDL, GLU, and ALT concentrations were higher in SLB pigs than in the DLY pigs (*p* < 0.05) in fattening stage 1. Compared to the DLY pigs, the levels of TG, AST, BUN, and TP in SLB pigs were reduced during the second fattening stage. The effects of dietary fiber level on serum biochemical parameters of finishing pigs were significant in fattening stage 1 (*p* < 0.05). The levels of GLU exhibited a decrease in response to increasing dietary fiber levels (*p* < 0.05). Group A exhibited significantly lower AST compared to the other dietary fiber groups (*p* < 0.05). The results of comparison among eight treatment groups showed that the fiber level and breed had significant effects on the levels of GLU, TG, AST, and ALT in the serum of fattening stage 1 (*p* < 0.05). Dietary fiber level and breed had significant interaction effects on serum GLU, AST, ALT, BUN, and TP levels in fattening stage 1 (*p* < 0.05).

### 3.4. Effects of Fiber Level on Cecal Volatile Fatty Acid in Different Breeds of Finishing Pigs

The effects of dietary fiber level and breed on the cecal VFA concentration of finishing pigs are presented in Table 6. In fattening stage 1, compared with DLY pigs, the concentration of propionate in the cecal digesta of SLB pigs was higher (*p* < 0.01), but the concentration of butyrate was lower (*p* < 0.05). In fattening stage 2, the concentrations of propionate and total volatile fatty acid (TVFA) of SLB pigs were lower than those of DLY pigs (*p* < 0.05). The concentrations of acetate, propionate, and TVFA in group B during fattening stage 1 were significantly higher under the influence of the dietary fiber level (*p* < 0.05). Additionally, acetate and TVFA concentrations during fattening stage 2 were also highest in group B (*p* < 0.05). In addition, the butyrate concentration in group C was lower than that in groups B and D in fattening stages 1 and 2 (*p* < 0.01). In fattening stage 2, the acetate concentration was significantly lower in the DD, SA, and SD groups compared to the DC, SB, and DB groups (*p* < 0.05). The propionate concentration was also significantly lower in the SC, SA, and DD groups compared to the SB, DA, DC, and DB groups (*p* < 0.05). Additionally, the butyrate concentration in pigs from the SD group was significantly lower than that in other dietary treatment groups (*p* < 0.05). Furthermore, the cecal TVFA concentration in DB, SB, DC, and DA groups was significantly higher than that in DD, SA, and SD groups (*p* < 0.05). It was observed that fiber level and breed had interaction effects on cecal VFA in fattening stage 1 as well as on cecal propionate and TVFA in fattening stage 2 of finishing pigs (*p* < 0.05).

### 3.5. Effects of Fiber Level on Small Intestine Morphology in Different Breeds of Finishing Pigs

The effects of dietary fiber level and breed on small intestine tissue morphology of finishing pigs are shown in Table 7. The results of the comparison among different breeds showed that the villus height and the ratio of the villus height to the crypt depth (V/C ratio) of the duodenum (fattening stage 1) and jejunum (fattening stage 2) were higher (*p* < 0.05) in DLY pigs than in SLB pigs, whereas the villus height of the jejunum (fattening stage 1) and the crypt depth of the duodenum (fattening stage 2) were lower (*p* < 0.05) in DLY pigs than in SLB pigs. Dietary fiber level had significant effects on villus height and crypt depth of the jejunum, as well as on the crypt depth of the ileum (fattening stage 1), duodenal V/C ratio, and villus height of the ileum (fattening stage 2) with different genetic backgrounds (*p* < 0.05). In fattening stage 1, the villus height of the jejunum in group C was lower than that in groups A and B (*p* < 0.05), while the crypt depth of the jejunum in group B was higher than that in the other three fiber level groups (*p* < 0.05). Meanwhile, the ileal crypt depth in group D was higher than that of groups A and B (*p* < 0.05). In fattening stage 2, the V/C ratio of the duodenum in group D was higher than that of groups A and C (*p* < 0.05). The villus height of the ileum in group A was higher than that of groups B and D (*p* < 0.05). The comparison results of the eight dietary treatment groups revealed that both the fiber level and genetic background significantly influenced the duodenal villus height and V/C ratio in fattening stage 1, as well as the duodenal villus height and crypt depth, and the jejunal and ileal villus height and crypt depth in fattening stage 2 (*p* < 0.05). The interaction effect of dietary fiber level and breed was observed on the duodenal villus height and V/C ratio of pigs in fattening stage 1, as well as on the duodenal villus height in fattening stage 2 (*p* < 0.05).

### 3.6. Effects of Fiber Level on Slaughtering Performance in Different Breeds of Finishing Pigs

The effects of fiber level and breed on the slaughter performance of finishing pigs are shown in Table 8. The comparison results among different breeds show that the backfat thickness of SLB pigs is higher than that of the DLY pigs in fattening stage 1 (*p* < 0.01), while the skin thickness is lower than that of the DLY pigs (*p* < 0.01). The fiber level has a significant effect on the loin eye area (LA), backfat thickness (fattening stage 1), and skin thickness (fattening stage 2) of finishing pigs with different genetic backgrounds. In fattening stage 1, pigs in group B have a higher LA and lower backfat thickness (*p* < 0.05) than the other dietary fiber level groups. At fattening stage 2, pigs in groups A and B had higher LA than groups C and D (*p* < 0.05). At the same time, the backfat thickness and skin thickness of group A are higher than those of the other three dietary fiber level groups (*p* < 0.05). The comparison results of the eight dietary treatment groups show that dietary fiber level and genetic background have a significant influence on the LA, backfat thickness, and skin thickness in fattening stages 1 and 2 (*p* < 0.05). Interaction between dietary fiber level and breed had a significant (*p* < 0.05) impact on the LA of pigs in fattening stage 1.

### 3.7. Effects of Fiber Level on Meat Quality in Different Breeds of Finishing Pigs

The analysis results of the relevant indicators for evaluating the quality of meat quality are shown in Table 9, Table 10 and Table 11.

The redness (a*) of SLB pigs was significantly higher than that of DLY pigs in the fattening stages (*p* < 0.05). The shear force of SLB pigs exceeded that of DLY pigs in fattening stage 2 (*p* < 0.05). The fiber level exhibited significant impacts on the pH_24h_, drip loss, lightness (L*), yellowness (b*), and shear force of muscle across different breeds (*p* < 0.05). During fattening stages 1 and 2, group A exhibited a higher drip loss compared to other fiber level groups (*p* < 0.05), whereas group B demonstrated a lower drip loss rate than groups C and D (*p* < 0.05). The pH_24h_ in group A was lower than that in group D in fattening stage 1 (*p* < 0.05). In fattening stage 2, the L* in group B exhibited a statistically significant decrease compared to groups A and C (*p* < 0.05), while the b* was also significantly lower than that in group D (*p* < 0.05). Additionally, the shear force in group A demonstrated a statistically significant increase compared to other dietary fiber level groups (*p* < 0.05). The interaction effects of dietary fiber level and breed were observed on the a* and drip loss of pigs in fattening stages 1 and 2, as well as on the L* and b* in fattening stage 2 (*p* < 0.05; Table 9).

The impact of different breeds on the nutrient composition of the LT is presented in Table 10. In stage 1, the levels of CP and EE in the LT were found to be significantly higher in SLY pigs than those in DLY pigs (*p* < 0.05). When the dietary fiber level was altered, the EE content of group B was higher than that of other groups in fattening stage 1 (*p* < 0.05). The EE content of group A was higher than that of groups C and D (*p* < 0.05), while the crude ash content of group A was lower than that of groups C and D (*p* < 0.05). The comparison results of eight treatment groups revealed that the levels of dietary fiber and the breed had significant impacts on the EE and crude ash contents of muscles during fattening stages 1 and 2 (*p* < 0.05). In stage 1, the EE content in the SB group was significantly higher than in other treatment groups (*p* < 0.05), while the crude ash content in the DA group was notably lower than in the SD, DC, and DD groups (*p* < 0.05). In stage 2, the EE content of the DD group was lower than that of the SA and SB groups (*p* < 0.05), with a similar trend observed for ash content compared to the SB, SC, and DB groups (*p* < 0.05). Furthermore, there was an interaction effect between dietary fiber level and breed on EE content during fattening stage 1 (*p* < 0.05).

The influence of dietary fiber level and breed on the LT fatty acid composition in finishing pigs is shown in Table 11. Comparative results across different breeds indicate that the SFA and PUFA content in SLB pigs is significantly higher than that in DLY pigs during fattening stage 2 (*p* < 0.05), while the MUFA content is notably lower than that in DLY pigs during both fattening stages (*p* < 0.05). Under the dietary fiber level factor, the SFA content of the LT in group D was lower than that in other fiber level groups (*p* < 0.05), and the MUFA content was higher than that in other fiber level groups during fattening stage 1 (*p* < 0.05). The PUFA content in the LT of pigs in group B was lower than that in group A during fattening stage 1 (*p* < 0.05). During fattening stage 2, the SFA content in the LT of pigs in groups A and B was higher than that in groups C and D (*p* < 0.05). The MUFA content in group B was lower than that in groups C and D (*p* < 0.05), but there was no difference compared with group A (*p* > 0.05). Additionally, the PUFA content in group B was higher than that in group A (*p* < 0.05), but there were no differences found in groups C and D (*p* > 0.05). There were no differences among the eight dietary treatment groups (*p* > 0.05). The interaction effect of dietary fiber level and breed on the fatty acid composition of the LT was significant (*p* < 0.05) (excluding C12:0, SFA, and MUFA at stage 1, as well as C18:0, C18:3n3, C24:1, SFA, and MUFA at stage 2).

## 4. Discussion

The utilization efficiency of fibrous agricultural by-products in the pig industry is influenced by factors such as fiber level and source, as well as the physiological stage of the pigs. The effects of various fiber sources or levels on pig performance at different physiological stages have been widely reported; for example, Liu et al. found that there is a difference in dietary fiber utilization by the two pig breeds (Taoyuan and Duroc), and the results might be associated with the integrity of the intestinal epithelium and the microbial activity of the gastrointestinal tract [20]. However, there are few comparative studies on the effects of fiber levels on the local breed (SLB) and commercial breed (DLY) of pigs. Further exploration of the interaction effects between different genetic backgrounds (breeds) and fiber levels on pig productivity is of great significance for the efficient use of fibrous feeds and the optimization of fiber nutrition requirements for different pig breeds. Therefore, the effects of dietary fiber level on the performance, nutrient utilization, intestinal development, and meat quality of SLB pigs and DLY pigs were investigated using fermented corn straw as a source of fiber.

This study found that DLY pigs have a greater growth advantage over SLB pigs throughout the entire fattening period. The appropriate dietary fiber level (CF: 4.82%) increased the FW and ADG of SLB pigs and DLY pigs during fattening stages 1 and 2, which was consistent with previous reports [21]. At the same time, the increase in dietary fiber levels improved the ADFI and F/G of SLB pigs and DLY pigs during the fattening period. The reduction in dietary CP and DE could be attributed to the higher proportion of fermented corn straw used as a replacement in this study, as pigs tend to increase their feed intake in order to meet their maintenance and production requirements [22]. The FW and ADG (fattening stages 1 and 2) in the SC and SD groups did not show significant differences compared to those in the SA group, whereas the FW and ADG (fattening stages 1 and 2) in the DD group were significantly lower than those in the DA and DB groups. This suggests that SLB pigs demonstrate an increased ability to adapt to high-fiber diets. Furthermore, the F/G in the DD group was significantly higher than that of the SD group during fattening stage 2. This indicates that when more fibrous by-products (18% fermented corn straw) are included in the diet or when the fiber level is increased (CF: 9.01%) during the fattening period, SLB pigs exhibit higher feed conversion efficiency.

Improving feed efficiency plays an important role in enhancing pig production benefits and reducing environmental burden [23]. In this study, we found that SLB pigs had a better utilization rate of fiber components during the fattening period compared to DLY pigs, which is related to their excellent roughage tolerance and stress resistance [24]. Moreover, the EE digestibility of SLB pigs was lower than that of DLY pigs during fattening stage 1, with no significant difference during stage 2. This indicates that as the fattening period progresses, the disparity in conventional nutrient utilization efficiency between DLY pigs and SLB pigs will continue to diminish, while the differences in CF and ADF utilization efficiency will continue to increase. Increasing the dietary fiber level to 6.86% (with a 12% substitution of fermented corn straw) resulted in reduced digestibility of CP, EE, CF, and ADF in SLB pigs and DLY pigs (fattening stage 1), consistent with reports [25]. In contrast, when the dietary fiber level was 4.82% (with a 6% replacement of FCS), it improved the CP and EE digestibility in both SLB and DLY pigs in stage 1. This improvement may be attributed to the presence of beneficial probiotics and digestive enzymes in the FCS, which facilitate the degrade of proteins and fats [26]. Furthermore, the elevation of dietary fiber levels leads to a reduction in the digestibility of fiber in both breeds. Unlike previous studies [27], the increase in dietary fiber levels did not have a negative impact on CP digestibility (fattening stage 2). This suggests that the influence of dietary fiber on the utilization efficiency of CP and EE in finishing pigs is dependent on the fattening stage. Therefore, when increasing the fiber level in the diet of finishing pigs (increasing the proportion of fibrous feed in the diet) within an appropriate range, attention should be given to the overall benefits during later stages or throughout the entire fattening period. Compared with DLY pigs, increasing dietary fiber levels did not reduce the CP digestibility of SLB pigs, which was consistent with the results of the growth performance of SLB pigs in this study. The interaction between dietary fiber level and genetic background has an impact on the digestibility of DM and CP in finishing pigs, indicating that both dietary fiber level and breed differences can influence the efficiency of DM and CP utilization in finishing pigs.

Research indicates that BUN and TP are closely related to protein absorption and utilization [28], while lipid metabolism indicators such as GLU, TG, LDL, HDL, and TC are related to pig fat deposition and metabolism [29]. Our findings show that blood LDL (fattening stages 1 and 2), GLU (fattening stage 1), and TG (fattening stage 2) in SLB pigs were higher than in DLY pigs, which may help explain the increased SFA and PUFA content in the LT of SLB pigs. This is because increased levels of LDL and TG in the blood can promote body fat synthesis, particularly SFA [29]. Meanwhile, higher GLU levels can stimulate insulin secretion, promoting the liver to convert excess glucose into fatty acid, especially SFA, thereby affecting fatty acid composition in the meat [30]. An increase in dietary fiber levels reduced GLU and TG content in the blood of both SLB and DLY pigs, which may be related to the increased viscosity and oil-holding capacity of the diet with higher fiber levels, thereby slowing the diffusion of glucose and free fatty acid to the intestinal epithelium [31,32]. Furthermore, an increase in dietary fiber levels did not negatively impact protein metabolism-related indicators in fattening pigs, which aligns with the CP digestibility results in the later fattening stages.

Dietary fiber is fermented by microorganisms in the large intestine of pigs to produce VFA such as acetate, propionate, and butyrate. Their production and proportion depend on the baseline microbial structure and the type of substrate. Research indicates that acetate affects host energy metabolism by reducing fat degrade and the number of pro-inflammatory cytokines, as well as by increasing energy expenditure and fat oxidation [33]. Propionate serves as a substrate for hepatic gluconeogenesis, participates in the body’s metabolic processes [34], and improves energy homeostasis through intestinal gluconeogenesis by converting to glucose [35,36]. Studies have shown that VFA content in pig digesta is positively correlated with dietary fiber levels [37], consistent with the results observed in this study. This study found that the highest VFA production in the cecum of both SLB and DLY pigs occurred at a dietary fiber level of 4.82%, which corresponds with the fiber component digestibility results.

Slaughter performance is an important indicator for evaluating the fattening efficiency of pigs, primarily including slaughter rate, lean meat percentage, LA, and backfat thickness. Generally, LA size is positively correlated with lean meat percentage, while backfat thickness is negatively correlated with it [38]. Previous studies have found that adding fermented feed or appropriately increasing fiber levels in the diet can improve carcass traits in fattening pigs [39,40,41]. The study by Coble et al. indicated that replacing corn–soybean meal diets with fiber-rich agricultural by-products (increasing NDF% from 7.35% to 14.95%) did not affect the LA and backfat thickness of pigs, which is inconsistent with our results (in this study, the fiber level was 4.82% CF and 14.09% NDF when 6% fermented corn straw was used to replace the basal diet). This heterogeneity may be due to differences in the physicochemical properties of different fiber sources [42]. In this study, when the dietary fiber level was increased to 4.82% (with a 6% replacement by FCS), the LA of both SLB and DLY crossbred pigs was improved during fattening stage 1, which may be related to the increased protein utilization efficiency of these pigs at a fiber level of 4.82%, and is consistent with the growth performance results. Additionally, further increasing the dietary fiber level (to CF levels of 6.86% and 9.81%) did not negatively affect the LA of pigs in the first fattening stage but reduced it in the second fattening stage. Meanwhile, the increase in the dietary fiber level reduced the backfat thickness of both SLB and DLY pigs. This is generally consistent with the findings of Hong et al. [43], who found that appropriately lowering dietary fiber levels could increase the loin eye area and backfat thickness of pigs. Moreover, the study by Li et al. [44] found that high-fiber ramie powder in the diet could reduce the backfat thickness of fattening pigs. The results of this study demonstrate that adding FCS to the diet can significantly reduce pig backfat thickness, proving its role in decreasing subcutaneous fat deposition and improving carcass traits. Previous research has also found that feeding fattening pigs a high-fiber diet leads to a reduction in backfat thickness [45], which can partially explain our results. Notably, increasing the dietary fiber level from 2.92% to 9.81% did not negatively affect the slaughter rate of SLB and DLY.

Meat color is an important sensory indicator. The a* is generally positively correlated with meat quality, while L* and b* are negatively correlated with it. Drip loss and shear force reflect the water-holding capacity and tenderness of the meat and both negatively correlated with meat quality [46]. The pH value is related to the flavor of the meat, with the pH of pork typically decreasing to 5.6–6.0 within 24 h after slaughter. This study found that the a* of the LT in SLB pigs during the first fattening stage was higher than that of DLY pigs crossbred pigs, and the drip loss was lower, indicating that SLB pigs have better meat quality. Studies have shown that increasing the fiber level in the diet of finishing pigs can significantly increase the a* of the LT, thereby improving meat quality [44]. On the other hand, higher dietary fiber levels promote the conversion of glycolytic muscle fibers to oxidative muscle fibers in the LT of fattening pigs, and the content of oxidative muscle fibers is positively correlated with the a* value [44], indicating that appropriately increasing the fiber level in the diet of finishing pigs plays an important role in improving their meat quality. This aligns with the findings of this study, where increasing the dietary fiber level raised the pH_24h_ of the LT in SLB pigs and DLY pigs during the first fattening stage and decreased drip loss in both the first and second fattening stages and shear force in the second fattening stage. Moreover, we found that during the first fattening stage, the CP and EE content in the LT of SLB pigs was significantly higher than that in DLY pigs, while in fattening stage 2, there was no difference in the nutrient composition of the LT between the two groups. It may be because the protein and fat deposition efficiency of SLB pigs may gradually slow down with the extension of fattening time. The nutrient digestibility of DLY pigs gradually improves during the late fattening period due to their heterosis characteristics, leading to increased protein and fat deposition and consequently progressively improved meat quality. It is well known that fat content is one of the key indicators for evaluating meat quality as it can indirectly affect the tenderness, flavor, and juiciness of cooked meat. When the fat content in the LT falls below 2.0–2.5%, it can negatively impact meat quality [47]. This study found that increasing the dietary fiber level to 4.82% increased the EE content in the LT of both SLB and DLY pigs, indicating that appropriately increasing the dietary fiber level in fattening pig diets tends to direct more energy substances toward intramuscular fat synthesis rather than body fat (subcutaneous fat). Research shows that the fatty acid composition in the LT plays an important role in meat quality and the acceptability of meat products because it determines the nutritional value and oxidative stability of the muscle [33]. This study found significant differences in the composition of several fatty acids in the LT of SLB and DLY pigs. The SFA and PUFA content in the LT of SLB pigs was higher than that of DLY pigs, while the MUFA content was lower. Additionally, it is noteworthy that increasing dietary fiber levels could increase the MUFA content and decrease the SFA content in the LT of both SLB and DLY pigs. This suggests that using fermented corn straw as a fiber source to increase dietary fiber levels can regulate the fatty acid composition in the meat of fattening pigs, which is of great significance for producing healthier and higher-quality meat.

## 5. Conclusions

In summary, the dietary fiber level and genetic background (breed) have an interactive effect on the production performance and nutrient utilization of finishing pigs. DLY pigs have a greater growth advantage, while SLB pigs show stronger adaptability to high-fiber diets. Moreover, FCS can be used as a good source of dietary fiber for both SLB and DLY pigs, which can improve the growth performance, carcass traits, and meat quality of finishing pigs. However, attention should be paid to the inclusion ratio in the diet and the overall fiber level for different pig breeds.

## Figures and Tables

**Table 1:** Analyzed composition of fermented corn straw (as-fed basis, %).

Items	DM	CP	EE	Ash	CF	ADF	NDF
Corn straw	54.73 ± 0.33	4.89 ± 0.12	2.02 ± 0.18	6.63 ± 0.11	40.86 ± 2.51	53.06 ± 0.63	73.48 ± 1.84
Fermented corn straw	52.55 ± 0.36	5.08 ± 0.23	2.71 ± 0.22	5.91 ± 0.17	35.20 ± 2.91	45.06 ± 0.46	66.88 ± 1.17

DM = dry matter; CP = crude protein; EE = ether extract; Ash = ash; CF = crude fiber; ADF = acid detergent fiber; NDF = neutral detergent fiber.

**Table 2:** Ingredients and chemical composition of experimental diets ^a^ (as-fed basis,%).

Items	A	B	C	D
Ingredients				
Corn	73.00	68.48	64.07	59.77
Soybean meal	15.00	14.09	13.12	12.08
fermented corn straw	0.00	6.00	12.00	18.00
Wheat bran	5.00	4.70	4.37	4.03
Rapeseed meal	4.50	4.23	3.94	3.62
Limestone	0.50	0.50	0.50	0.50
Dicalcium phosphate	1.00	1.00	1.00	1.00
Vitamin and mineral premix ^b^	1.00	1.00	1.00	1.00
Total	100	100	100	100
Calculated nutrition content ^c^ (%, as DM)				
DE, MJ/kg	13.43	12.77	12.05	11.29
CP	14.78	14.22	13.63	13.00
CF	2.92	4.82	6.86	9.01
ADF	4.69	7.06	9.60	12.30
NDF	10.80	14.09	17.61	21.34
Ca	0.49	0.48	0.5	0.51
TP	0.46	0.46	0.46	0.45

^a^ A = diet with 2.92% crude fiber level; B = diet with 4.82% crude fiber level; C = diet with 6.86% crude fiber level; D = diet with 9.01% crude fiber level; DE = digestible energy; CP = crude protein; CF = crude fiber; ADF = acid detergent fiber; NDF = neutral detergent fiber; Ca = calcium; TP = total phosphorus. ^b^ Supplied the following per kilogram complete diet, vitamin A, 28,500 IU; vitamin D, 36,000 IU; vitamin E, 67.5 IU; vitamin K, 37.5 mg; vitamin B1, 17.5 mg; vitamin B2, 215 mg; vitamin B6, 69 mg; vitamin B12, 0.075 mg, nicotinic acid, 70 mg, folic acid, 3 mg, calcium pantothenate, 0.375 mg, antioxidant, 0.15 mg, choline chloride, 105 mg; Co as CoSO_4_, 1 mg; Cu as CuSO_4_·5H_2_O, 155 mg; Fe as FeSO_4_·H_2_O, 145 mg; Mn as MnO, 75 mg; Zn as ZnSO_4_, 125 mg; I as KI, 0.3 mg; Se as Na_2_SeO_3_, 0.3 mg. ^c^ Data are calculated according to NRC (2012) standards [14].

**Table 3:** Effects of dietary fiber level and breed on the growth performance of the finishing pigs.

Fattening Stage	Items	Breed		Crude Fiber Level (F)				Treatment								SEM	*p* Value		
		S (SLB)	D (DLY)	A (2.92%)	B (4.82%)	C (6.86%)	D (9.01%)	SA	SB	SC	SD	DA	DB	DC	DD		G	F	G × F
Fattening stage 1	IW, kg	60.55	60.87	60.61	60.79	60.62	60.83	60.31	60.82	60.39	60.69	60.91	60.76	60.84	60.96	0.01	0.51	0.48	0.35
	FW, kg	85.68 ^b^	87.89 ^a^	87.34 ^b^	89.31 ^a^	86.39 ^b^	84.10 ^c^	85.48 ^bc^	87.65 ^abc^	85.26 ^bc^	84.34 ^bc^	89.20 ^ab^	90.96 ^a^	87.52 ^abc^	83.86 ^c^	0.59	0.03	<0.01	<0.01
	ADG, kg	0.65 ^b^	0.69 ^a^	0.69 ^ab^	0.71 ^a^	0.67 ^b^	0.60 ^c^	0.65 ^cde^	0.68 ^bcd^	0.64 ^de^	0.61 ^ef^	0.73 ^ab^	0.75 ^a^	0.69 ^abc^	0.58 ^f^	0.01	<0.01	<0.01	0.02
	ADFI, kg	2.48 ^b^	2.58 ^a^	2.22 ^d^	2.47 ^c^	2.63 ^b^	2.80 ^a^	2.19 ^d^	2.44 ^c^	2.54 ^bc^	2.75 ^a^	2.25 ^d^	2.51 ^c^	2.71 ^ab^	2.85 ^a^	0.05	0.03	<0.01	0.77
	F/G	3.99	3.83	3.24 ^d^	3.51 ^c^	4.12b	4.78 ^a^	3.40 ^de^	3.62 ^d^	4.27 ^b^	4.70 ^a^	3.09 ^e^	3.40 ^de^	3.97 ^c^	4.86 ^a^	0.06	0.27	<0.01	0.10
Fattening stage 2	IW, kg	85.66 ^b^	87.90 ^a^	87.33 ^b^	89.34 ^a^	86.38 ^b^	84.09 ^c^	85.42 ^bc^	87.68 ^abc^	85.23b ^c^	84.32 ^bc^	89.24 ^ab^	90.99 ^a^	87.53 ^abc^	83.85 ^c^	0.59	0.02	<0.01	<0.01
	FW, kg	109.12 ^b^	112.72 ^a^	112.53 ^b^	115.56 ^a^	109.94 ^c^	105.67 ^c^	109.03 ^cd^	112.43 ^bc^	108.64 ^cd^	106.38 ^d^	116.02 ^ab^	118.68 ^a^	111.23 ^c^	104.96 ^d^	0.67	<0.01	<0.01	<0.01
	ADG, kg	0.59 ^b^	0.63 ^a^	0.65 ^a^	0.66 ^a^	0.59 ^b^	0.55 ^c^	0.61 ^b^	0.62 ^b^	0.58 ^bc^	0.56 ^c^	0.69 ^a^	0.70 ^as^	0.61 ^b^	0.54 ^c^	0.01	<0.01	<0.01	<0.01
	ADFI, kg	2.76 ^b^	3.00 ^a^	2.63 ^c^	2.85 ^b^	2.99 ^ab^	3.04 ^a^	2.50 ^f^	2.69 ^e^	2.87 ^d^	2.96 ^cd^	2.75 ^e^	3.02 ^b^	3.11 ^abc^	3.12 ^a^	0.04	<0.01	<0.01	0.09
	F/G	4.74	4.80	4.12^d^	4.39^c^	5.09 ^b^	5.48 ^a^	4.22 ^cd^	4.38^c^	5.06 ^b^	5.29 ^b^	4.02 ^d^	4.39^c^	5.13 ^b^	5.66 ^a^	0.06	0.46	<0.01	0.09

S = Songliao black; D = Duroc × Landrac × Yorkshire; A = diet with 2.92% crude fiber level; B = diet with 4.82% crude fiber level; C = diet with 6.86% crude fiber level; D = diet with 9.01% crude fiber level; G = genetic basis (breed); F = crude fiber level; G × F = interaction effect between crude fiber level and breed; IW = initial weight; FW = final weight; ADG = average daily gain; ADFI = average daily feed intake; F/G = ratio of feed to weight gain. Means within the same row for the individual effects of crude fiber level, genetic basis (breed), and dietary treatments with different superscript letters differ (*p* < 0.05).

**Table 4:** Effects of dietary fiber level and breed on nutrient digestibility of finishing pigs. (%, apparent total tract digestibility).

Fattening Stage	Items	Breed		Crude Fiber Level (F)				Treatment								SEM	*p* Value		
		S (SLB)	D (DLY)	A (2.92%)	B (4.82%)	C (6.86%)	D (9.01%)	SA	SB	SC	SD	DA	DB	DC	DD		G	F	G × F
Fattening stage 1	DM	80.82	80.40	83.74 ^a^	81.91 ^b^	79.35 ^c^	77.46 ^d^	82.99 ^b^	81.49 ^c^	79.99 ^d^	78.82 ^d^	84.49 ^a^	82.32 ^bc^	78.70 ^d^	76.09 ^e^	0.34	0.22	<0.01	<0.01
	CP	76.60	76.55	77.89 ^a^	77.77 ^a^	75.32 ^b^	75.33 ^b^	77.26 ^ab^	77.18 ^abc^	75.12 ^cd^	76.83 ^abc^	78.52 ^a^	78.36 ^a^	75.51 ^bcd^	73.82 ^d^	0.30	0.93	<0.01	<0.01
	EE	62.68 ^b^	65.16 ^a^	65.03 ^ab^	65.84 ^a^	62.06 ^b^	62.75 ^ab^	62.85 ^c^	64.00 ^abc^	60.93 ^c^	62.96 ^c^	67.22 ^ab^	67.68 ^a^	63.20 ^bc^	62.54 ^c^	0.53	0.01	0.01	0.395
	CF	46.42 ^a^	43.33 ^b^	49.05 ^a^	45.43 ^b^	43.39 ^bc^	41.64 ^c^	50.35 ^a^	47.24 ^ab^	44.68 ^bc^	43.41 ^c^	47.76 ^ab^	43.63 ^c^	42.10 ^cd^	39.86 ^d^	0.53	<0.01	<0.01	0.937
	ADF	47.16 ^a^	45.14 ^b^	49.77 ^a^	49.93 ^a^	46.09 ^b^	38.80 ^b^	50.26 ^a^	50.73 ^a^	46.69 ^bc^	40.97 ^d^	49.28 ^ab^	49.13 ^ab^	45.50 ^c^	36.64 ^e^	0.70	<0.01	< 0.01	0.34
	NDF	44.60 ^a^	42.46 ^b^	46.31 ^a^	45.68 ^a^	42.35 ^b^	39.80 ^c^	46.35 ^a^	46.09 ^a^	43.91 ^ab^	42.07 ^bc^	46.27 ^a^	45.26 ^a^	40.79 ^c^	37.53 ^d^	0.48	<0.01	< 0.01	0.059
Fattening stage 2	DM	84.56	85.53	85.54 ^ab^	86.27 ^a^	84.97 ^b^	83.39 ^c^	85.6	85.87	83.80	82.97	85.48	86.66	85.95	83.82	0.18	0.06	< 0.01	< 0.01
	CP	82.82	83.99	82.11 ^b^	85.02 ^a^	84.12 ^a^	82.37 ^b^	82.25 ^bc^	84.52 ^a^	82.72 ^bc^	81.79 ^c^	81.97 ^bc^	85.53 ^a^	85.53 ^a^	82.95 ^b^	0.40	<0.01	< 0.01	< 0.01
	EE	68.57	71.75	70.23	72.05	69.05	69.32	69.52 ^ab^	70.85 ^ab^	65.57 ^c^	68.33 ^bc^	70.94 ^ab^	73.25 ^a^	72.52 ^ab^	70.31 ^ab^	0.49	<0.01	0.06	0.08
	CF	53.28 ^a^	47.91 ^b^	55.78 ^a^	51.24 ^b^	48.56 ^bc^	46.81 ^c^	57.39 ^a^	54.63 ^ab^	51.21 ^cd^	49.91 ^de^	54.17 ^bc^	47.85 ^ef^	45.92 ^fg^	43.70 ^g^	0.63	<0.01	< 0.01	0.37
	ADF	59.17 ^a^	53.91 ^b^	61.83 ^a^	60.86 ^a^	55.16 ^b^	48.33 ^c^	64.41 ^a^	63.55 ^a^	57.42 ^b^	49.93 ^c^	59.25 ^b^	58.16 ^b^	52.90 ^c^	45.35 ^d^	0.82	<0.01	< 0.01	0.932
	NDF	54.94	54.19	59.31 ^a^	57.72 ^a^	50.98 ^b^	50.27 ^b^	60.15 ^a^	57.62 ^a^	51.10 ^b^	50.90 ^b^	58.46 ^a^	57.83 ^a^	50.85 ^b^	49.63 ^b^	0.59	0.27	< 0.01	0.76

S = Songliao black; D = Duroc × Landrac × Yorkshire; A = diet with 2.92% crude fiber level; B = diet with 4.82% crude fiber level; C = diet with 6.86% crude fiber level; D = diet with 9.01% crude fiber level; G = genetic basis (breed); F = crude fiber level; G × F = interaction effect between crude fiber level and breed; DM = dry matter; CP = crude protein; EE = ether extract; CF = crude fiber; ADF = acid detergent fiber; NDF = neutral detergent fiber. Means within the same row for the individual effects of crude fiber level, genetic basis (breed), and dietary treatments with different superscript letters differ (*p* < 0.05).

**Table 5:** Effects of dietary fiber level and breed on serum biochemical parameters of finishing pigs.

Fattening Stage	Items	Breed		Crude Fiber Level (F)				Treatment								SEM	*p* Value		
		S (SLB)	D (DLY)	A (2.92%)	B (4.82%)	C (6.86%)	D (9.01%)	SA	SB	SC	SD	DA	DB	DC	DD		G	F	G × F
Fattening stage 1	LDL, mm/L	1.31 ^a^	1.22 ^b^	1.22	1.26	1.27	1.31	1.22 ^ab^	1.28 ^ab^	1.40 ^a^	1.35 ^a^	1.22 ^ab^	1.24 ^ab^	1.15 ^b^	1.28 ^ab^	0.02	0.04	0.44	0.22
	HDL, mm/L	1.15	1.06	1.07	1.04	1.20	1.12	1.10	1.02	1.24	1.24	1.04	1.05	1.16	1.00	0.32	0.19	0.36	0.51
	GLU, mm/L	5.04 ^a^	4.82 ^b^	5.24 ^a^	5.05 ^ab^	4.80 ^bc^	4.64 ^c^	5.91 ^a^	5.03 ^bc^	4.86 ^b^	4.35 ^d^	4.57 ^cd^	5.07 ^b^	4.73 ^bcd^	4.93 ^bc^	0.09	0.04	<0.01	<0.01
	TG, mm/L	0.50	0.72	0.69 ^a^	0.62 ^ab^	0.56 ^b^	0.58 ^b^	0.56 ^cd^	0.53 ^d^	0.43 ^d^	0.49 ^d^	0.82 ^a^	0.71 ^ab^	0.69 ^abc^	0.67 ^bc^	0.03	<0.01	0.04	0.67
	TC, mm/L	2.29	2.25	2.21	2.24	2.37	2.25	2.21	2.24	2.39	2.3	2.21	2.32	2.34	2.21	0.03	0.62	0.54	0.97
	AST, U/L	42.83	37.75	34.93 ^c^	41.29 ^b^	39.48 ^b^	45.45 ^a^	31.90 ^e^	47.03 ^a^	42.07 ^b^	50.30 ^a^	37.95 ^bcd^	36.90 ^de^	36.90 ^cd^	40.60 ^bc^	1.19	<0.01	<0.01	<0.01
	ALT, U/L	54.82 ^a^	50.73 ^b^	49.18 ^b^	53.01 ^b^	58.62 ^a^	50.29 ^b^	49.67 ^c^	51.37 ^bc^	60.10 ^a^	58.15 ^ab^	48.70 ^cd^	54.65 ^abc^	57.13 ^ab^	42.43 ^d^	1.26	0.01	<0.01	<0.01
	BUN, mm/L	6.80	6.85	7.25	6.47	7.02	6.58	7.23 ^ab^	6.13 ^bc^	6.10 ^bc^	7.75 ^a^	7.28 ^ab^	6.80 ^ab^	5.40 ^c^	7.93 ^a^	0.20	0.86	0.14	<0.01
	TP, g/L	71.85 ^b^	74.14 ^a^	73.10	72.28	73.12	73.49	72.37 ^bc^	72.50 ^bc^	73.17 ^bc^	69.38 ^c^	73.83 ^b^	72.00 ^bc^	73.07 ^bc^	77.60 ^a^	0.57	0.01	0.74	<0.01
	ALB, g/L	45.12	44.64	45.04	44.35	44.78	45.34	46.73	44.03	45.2	44.53	43.35	44.68	44.37	46.15	0.61	0.72	0.96	0.58
Fattening stage 2	LDL, mm/L	1.16 ^a^	0.96 ^b^	1.10	0.93	1.17	1.04	1.09 ^ab^	1.11 ^ab^	1.37 ^a^	1.06 ^abc^	1.10 ^a^	0.76 ^c^	0.98 ^bc^	1.02 ^bc^	0.04	0.01	0.14	0.13
	HDL, mm/L	1.08	1.03	1.09	1.01	1.10	1.03	1.15	0.96	1.16	1.05	1.03	1.06	1.03	1.00	0.03	0.43	0.63	0.52
	GLU, mm/L	4.71	4.56	4.70	4.52	4.41	4.90	4.76	4.51	4.6	4.97	4.64	4.53	4.22	4.84	0.08	0.36	0.16	0.85
	TG, mm/L	0.47 ^b^	0.63 ^a^	0.58	0.62	0.50	0.49	0.51 ^bc^	0.47 ^bc^	0.45 ^bc^	0.40 ^c^	0.64 ^ab^	0.74 ^a^	0.55 ^abc^	0.58 ^abc^	0.03	0.01	0.24	0.78
	TC, mm/L	2.11	1.98	2.07	2.00	2.13	1.98	2.15 ^ab^	1.98 ^ab^	2.35 ^a^	1.97 ^ab^	2.00 ^ab^	2.03 ^ab^	1.92 ^b^	1.98 ^ab^	0.04	0.15	0.59	0.24
	AST, U/L	32.92 ^a^	35.62^b^	32.33 ^b^	34.74 ^ab^	36.13 ^a^	33.88 ^ab^	31.20 ^b^	33.90 ^ab^	33.87 ^ab^	32.70 ^b^	33.46 ^ab^	35.58 ^ab^	38.40 ^a^	35.05 ^ab^	0.62	0.03	0.15	0.83
	ALT, U/L	53.55	54.73	50.25	54.09	53.88	58.34	51.40 ^b^	53.63 ^b^	55.30 ^b^	53.88 ^b^	49.10 ^b^	54.55 ^b^	52.45 ^b^	62.80 ^a^	0.96	0.48	0.02	0.07
	BUN, mm/L	5.77 ^b^	6.68 ^a^	6.62	5.74	6.22	6.33	6.13 ^abc^	5.40 ^c^	5.77 ^bc^	5.80 ^bc^	7.10 ^a^	6.08 ^abc^	6.68 ^ab^	6.85 ^ab^	0.15	<0.01	0.94	0.15
	TP, g/L	69.56 ^b^	73.43 ^a^	73.37	71.78	70.68	70.16	70.27 ^bc^	70.08 ^bc^	70.30 ^bc^	67.60 ^c^	76.48 ^a^	73.48 ^ab^	71.05 ^bc^	72.73 ^abc^	0.70	<0.01	0.24	0.41
	ALB, g/L	43.87	45.56	43.93	42.73	46.09	46.11	44.60 ^ab^	40.48 ^b^	45.60 ^ab^	44.80 ^ab^	43.26 ^ab^	44.98 ^ab^	46.58 ^a^	47.43 ^a^	0.65	0.19	0.17	0.41

S = Songliao black; D = Duroc × Landrac × Yorkshire; A = diet with 2.92% crude fiber level; B = diet with 4.82% crude fiber level; C = diet with 6.86% crude fiber level; D = diet with 9.01% crude fiber level; G = genetic basis (breed); F = crude fiber level; G × F = interaction effect between crude fiber level and breed; LDL = low-density lipoprotein; HDL = high-density lipoprotein; GLU = blood glucose; TG = triglyceride; TC = total cholesterol; AST = aspartate aminotransferase; ALT = alanine aminotransferase; BUN = blood urea nitrogen; TP = total protein; ALB = albumin. Means within the same row for the individual effects of crude fiber level, genetic basis (breed), and dietary treatments with different superscript letters differ (*p* < 0.0).

**Table 6:** Effects of dietary fiber level and breed on volatile fatty acid in cecum of finishing pigs. (µmol/g, fresh sample-basis).

Fattening Stage	Items	Breed		Crude Fiber Level (F)				Treatment								SEM	*p* Value		
		S (SLB)	D (DLY)	A (2.92%)	B (4.82%)	C (6.86%)	D (9.01%)	SA	SB	SC	SD	DA	DB	DC	DD		G	F	G × F
Fattening stage 1	Acetate	66.29	67.62	63.92 ^b^	80.69 ^a^	62.91 ^b^	60.31 ^b^	56.68 ^c^	86.91 ^a^	63.08 ^c^	58.47 ^c^	71.16 ^b^	74.46 ^b^	62.73 ^c^	62.14 ^c^	2.08	0.44	<0.01	<0.01
	Propionate	21.78 ^a^	17.99 ^b^	19.63 ^b^	22.21 ^a^	18.03 ^b^	19.68 ^b^	17.23 ^de^	25.58 ^a^	21.33 ^bc^	23.00 ^ab^	22.02 ^bc^	18.84 ^cd^	14.73 ^e^	16.36 ^de^	0.78	<0.01	<0.01	<0.01
	Butyrate	5.16 ^b^	5.62 ^a^	5.20 ^ab^	6.33 ^a^	4.15 ^b^	5.89 ^a^	4.32 ^cd^	7.77 ^a^	3.76 ^d^	4.79 ^c^	6.06 ^b^	4.88 ^c^	4.53 ^cd^	6.99 ^a^	1.41	0.04	<0.01	<0.01
	TVFA	93.23	91.22	88.74 ^b^	109.22 ^a^	85.08 ^b^	85.87 ^b^	78.23 ^c^	120.26 ^a^	88.17 ^c^	86.26 ^c^	99.24 ^b^	98.18 ^b^	81.99 ^c^	85.48 ^c^	2.78	0.39	<0.01	<0.01
Fattening stage 2	Acetate	76.22	78.17	75.27 ^bc^	83.50 ^a^	78.44 ^b^	71.58 ^c^	72.78 ^cd^	82.48 ^ab^	76.73 ^bc^	72.88 ^cd^	77.76 ^bc^	84.52 ^a^	80.14 ^ab^	70.28 ^d^	1.12	0.15	<0.01	0.23
	Propionate	21.52 ^b^	23.46 ^a^	22.20 ^b^	23.89 ^a^	22.36 ^ab^	21.52 ^b^	20.30 ^b^	22.94 ^a^	20.22 ^b^	22.60 ^ab^	24.10 ^a^	24.83 ^a^	24.49 ^a^	20.43 ^b^	0.44	<0.01	0.04	<0.01
	Butyrate	8.35	8.09	8.86 ^ab^	9.55 ^a^	8.35 ^b^	6.12 ^c^	8.70 ^b^	9.83 ^a^	8.23 ^b^	6.64 ^c^	9.02 ^ab^	9.27 ^ab^	8.47 ^b^	5.60 ^d^	0.29	0.27	<0.01	0.14
	TVFA	106.08 ^b^	109.73 ^a^	106.33 ^b^	116.93 ^a^	109.14 ^b^	99.22 ^c^	101.79 ^de^	115.25 ^ab^	105.17 ^cd^	102.13 ^de^	110.88 ^bc^	118.62 ^a^	113.11 ^ab^	96.31 ^e^	1.65	0.04	0.02	0.02

S = Songliao black; D = Duroc × Landrac × Yorkshire; A = diet with 2.92% crude fiber level; B = diet with 4.82% crude fiber level; C = diet with 6.86% crude fiber level; D = diet with 9.01% crude fiber level; G = genetic basis (breed); F = crude fiber level; G × F = interaction effect between crude fiber level and breed; TVFA = total volatile fatty acid. Means within the same row for the individual effects of crude fiber level, genetic basis (breed), and dietary treatments with different superscript letters differ (*p* < 0.05).

**Table 7:** Effects of dietary fiber level and breed on intestinal morphology of finishing pigs.

Fattening Stage	Items		Breed		Crude Fiber Level (F)				Treatment								SEM	*p* Value		
			S (SLB)	D (DLY)	A (2.92%)	B (4.82%)	C (6.86%)	D (9.01%)	SA	SB	SC	SD	DA	DB	DC	DD		G	F	G × F
Fattening stage 1	Duodenum	Villus height, µm	970.43 ^b^	1043.01 ^a^	996.35	1020.48	1020.93	989.12	862.00 ^c^	904.70 ^bc^	1038.07 ^ab^	1076.97 ^a^	1130.70 ^a^	1136.27 ^a^	1003.80 ^abc^	901.27^bc^	24.86	0.04	0.85	0.01
		Crypt depth, µm	213.63	207.93	200.87	212.37	209.97	219.90	204.33	212.63	214.63	222.9	197.4	212.1	205.308	216.9	4.53	0.58	0.63	0.99
		V/C	4.55 ^b^	5.03 ^a^	4.88	4.84	4.91	4.51	4.17 ^b^	4.31 ^b^	4.84 ^ab^	4.87 ^ab^	5.38 ^a^	5.59 ^a^	4.97 ^ab^	4.16 ^b^	0.13	0.03	0.44	<0.01
	Jejunum	Villus height, µm	1101.72 ^a^	1025.98 ^b^	1112.86 ^a^	1112.15 ^a^	986.92 ^b^	1043.48 ^ab^	1129.35 ^ab^	1208.50 ^a^	1050.43 ^bc^	1018.60 ^bc^	1096.37 ^ab^	1015.80 ^bc^	923.40^c^	1068.35 ^ab^	20.46	0.03	0.03	0.07
		Crypt depth, µm	215.90	208.92	206.97 ^b^	228.35 ^a^	205.93 ^b^	208.38 ^b^	204.17 ^b^	237.07 ^a^	211.05 ^b^	211.30 ^b^	209.77 ^b^	219.63 ^ab^	200.80 ^b^	205.47 ^b^	3.24	0.20	0.02	0.44
		V/C	5.03	4.91	5.41 ^a^	4.78 ^b^	4.76 ^b^	4.94 ^ab^	5.59	4.92	4.88	4.74	5.24	4.63	4.64	5.13	0.11	0.55	0.12	0.56
	Ileum	Villus height, µm	915.23	912.57	989.75	874.63	878.78	912.43	1047.70 ^a^	813.30 ^b^	904.07 ^b^	895.80 ^b^	931.80 ^ab^	935.90 ^ab^	853.50 ^b^	929.07 ^ab^	16.87	0.93	0.05	0.06
		Crypt depth, µm	235.99	233.73	218.22^b^	215.92 ^b^	241.77 ^ab^	263.53 ^a^	222.90 ^ab^	213.00 ^b^	245.00 ^ab^	263.07 ^a^	213.53 ^b^	218.83 ^ab^	238.53 ^ab^	264.00 ^a^	5.68	0.82	0.01	0.94
		V/C	3.96	4.01	4.62 ^a^	4.12 ^ab^	3.64 ^b^	3.56 ^b^	4.81 ^a^	3.89 ^ab^	3.70 ^ab^	3.45 ^b^	4.43 ^ab^	4.36 ^ab^	3.59 ^ab^	3.68 ^ab^	0.14	0.84	0.05	0.70
Fattening stage 2	Duodenum	Villus height, µm	989.31	972.32	937.33 ^b^	996.83 ^ab^	955.60 ^ab^	1033.50 ^a^	951.77 ^ab^	1065.77 ^a^	930.00 ^b^	1009.70 ^ab^	922.90 ^b^	927.90 ^b^	981.20 ^ab^	1057.30 ^a^	15.29	0.50	0.06	0.04
		Crypt depth, µm	230.90 ^a^	213.57 ^b^	228.90	219.62	229.35	211.08	239.47 ^ab^	227.53 ^abc^	248.80 ^a^	207.80 ^c^	218.33 ^bc^	211.70 ^c^	209.90 ^c^	214.35 ^bc^	3.85	<0.01	0.12	0.10
		V/C	4.35	4.58	4.07 ^b^	4.55 ^ab^	4.26 ^b^	4.98 ^a^	3.98 ^cd^	4.70 ^abc^	3.87 ^d^	4.86 ^ab^	4.39 ^abcd^	5.10 ^a^	4.65 ^abc^	4.16 ^bcd^	0.11	0.18	0.01	0.18
	Jejunum	Villus height, µm	979.85 ^b^	1042.00 ^a^	1039.67	986.05	1032.27	985.72	967.73 ^c^	973.13 ^c^	994.80 ^bc^	983.73 ^bc^	1111.60 ^a^	998.97 ^bc^	1069.73 ^ab^	987.70 ^bc^	12.11	<0.01	0.11	0.09
		Crypt depth, µm	209.53	214.36	210.68	210.47	213.90	212.73	208.03 ^ab^	214.17 ^ab^	199.67 ^b^	216.27 ^ab^	213.32 ^ab^	206.77 ^ab^	228.13 ^a^	209.20 ^ab^	2.71	0.37	0.96	0.09
		V/C	4.69	4.90	4.97	4.69	4.85	4.67	4.65	4.55	4.98	4.58	5.29	4.83	4.70	4.77	0.789	0.20	0.53	0.29
	Ileum	Villus height, µm	982.24	1013.17	1073.53 ^a^	945.54 ^b^	974.33 ^ab^	997.40 ^ab^	1013.37 ^b^	924.95 ^b^	992.30 ^b^	998.33 ^b^	1133.70 ^a^	966.13 ^b^	956.37 ^b^	996.47 ^b^	15.69	0.28	0.04	0.27
		Crypt depth, µm	229.26	219.21	217.07 ^ab^	206.58 ^b^	238.10 ^a^	235.18 ^ab^	215.00 ^ab^	218.27 ^ab^	234.43 ^ab^	249.33 ^a^	219.13 ^ab^	194.90 ^b^	241.77 ^a^	221.03 ^ab^	5.21	0.31	0.10	0.46
		V/C	4.32	4.63	4.84	4.60	4.14	4.31	4.72	4.25	4.26	4.05	4.96	4.95	4.02	4.57	0.13	0.21	0.20	0.51

S = Songliao black; D = Duroc × Landrac × Yorkshire; A = diet with 2.92% crude fiber level; B = diet with 4.82% crude fiber level; C = diet with 6.86% crude fiber level; D = diet with 9.01% crude fiber level; G = genetic basis (breed); F = crude fiber level; G × F = interaction effect between crude fiber level and breed; V/C = ratio of villus height to crypt depth. Means within the same row for the individual effects of crude fiber level, genetic basis (breed), and dietary treatments with different superscript letters differ (*p* < 0.05).

**Table 8:** Effects of dietary fiber level and breed on slaughter traits of finishing pigs.

Fattening Stage	Items	Breed		Crude Fiber Level (F)				Treatment								SEM	*p* Value		
		S (SLB)	D (DLY)	A (2.92%)	B (4.82%)	C (6.86%)	D (9.01%)	SA	SB	SC	SD	DA	DB	DC	DD		G	F	G × F
Fattening stage 1	DP, %	63.55	63.81	63.32	64.03	63.04	64.33	62.67	63.95	62.67	64.9	63.97	64.11	63.4	63.75	0.38	0.77	0.70	0.78
	LA, cm^2^	43.18	42.47	42.18 ^b^	44.64 ^a^	41.92 ^b^	42.57 ^b^	44.17 ^ab^	45.37 ^a^	42.16 ^bcd^	41.03 ^cd^	40.19 ^d^	43.91 ^abc^	41.68 ^bcd^	44.10 ^ab^	0.44	0.28	0.03	0.01
	BT, mm	28.75 ^a^	23.92 ^b^	29.72 ^a^	24.43 ^b^	26.71 ^b^	24.49 ^b^	31.05 ^a^	29.33 ^ab^	29.04 ^abc^	25.57 ^abc^	28.39 ^abc^	19.53 ^d^	24.38 ^bcd^	23.40 ^cd^	0.90	<0.01	0.02	0.15
	ST, mm	2.98 ^b^	3.39 ^a^	3.33	3.05	2.97	3.39	3.09 ^abc^	2.73 ^c^	2.83 ^bc^	3.26 ^abc^	3.56 ^a^	3.38 ^ab^	3.11 ^abc^	3.53 ^a^	0.08	<0.01	0.08	0.66
Fattening stage 2	DP, %	68.90	69.54	69.60	69.60	69.17	68.52	70.02	69.80	68.65	67.15	69.19	69.39	69.69	69.89	0.34	0.37	0.66	0.29
	LA, cm^2^	45.54	47.48	48.82 ^a^	49.13 ^a^	43.37 ^b^	44.73 ^b^	47.51 ^abc^	47.23 ^abc^	43.55 ^c^	43.87 ^bc^	50.12 ^ab^	51.03 ^a^	43.18 ^c^	45.59 ^abc^	0.81	0.17	0.02	0.74
	BT, mm	28.78	27.27	32.84 ^a^	27.31 ^c^	23.37 ^d^	28.58 ^bc^	34.09 ^a^	29.18 ^bc^	24.19 ^de^	27.67 ^bcd^	31.59 ^ab^	25.43 ^cde^	22.55 ^e^	29.49 ^bc^	0.85	0.14	<0.01	0.25
	ST, mm	3.72	3.51	4.22 ^a^	3.55 ^b^	3.32 ^b^	3.35 ^b^	4.53 ^a^	3.47 ^bc^	3.55 ^bc^	3.31 ^c^	3.91 ^b^	3.63 ^bc^	3.10 ^c^	3.39 ^bc^	0.09	0.09	<0.01	0.07

S = Songliao black; D = Duroc × Landrac × Yorkshire; A = diet with 2.92% crude fiber level; B = diet with 4.82% crude fiber level; C = diet with 6.86% crude fiber level; D = diet with 9.01% crude fiber level; G = genetic basis (breed); F = crude fiber level; G × F = interaction effect between crude fiber level and breed; DP = dressing percentage; LA = Loin eye area; BT = backfat thickness; ST = skin thickness. Means within the same row for the individual effects of crude fiber level, genetic basis (breed), and dietary treatments with different superscript letters differ (*p* < 0.05).

**Table 9:** Effects of dietary fiber level and breed on meat quality of finishing pigs.

Fattening Stage	Items	Breed		Crude Fiber Level (F)				Treatment								SEM	*p* Value		
		S (SLB)	D (DLY)	A (2.92%)	B (4.82%)	C (6.86%)	D (9.01%)	SA	SB	SC	SD	DA	DB	DC	DD		G	F	G × F
Fattening Stage 1	pH_24h_	5.66	5.67	5.61 ^b^	5.69 ^ab^	5.64 ^ab^	5.72 ^a^	5.61 ^b^	5.69 ^ab^	5.69 ^ab^	5.66 ^ab^	5.62 ^b^	5.69 ^ab^	5.59 ^b^	5.78 ^a^	0.01	0.86	0.04	0.05
	L*	51.86	52.85	53.32	51.84	51.90	52.35	51.52	51.76	50.56	53.59	55.12	51.91	53.24	51.11	0.56	0.40	0.76	0.28
	a*	9.41 ^a^	7.41 ^b^	8.74	8.65	7.99	8.26	10.59 ^a^	9.74 ^a^	10.23 ^a^	9.08 ^a^	6.90 ^b^	7.56 ^b^	5.74 ^c^	9.43 ^a^	0.39	<0.01	0.195	<0.01
	b*	14.89	14.86	15.64	14.40	15.10	14.37	15.81	14.32	15.80	13.64	15.47	14.48	14.39	15.11	0.26	0.95	0.24	0.26
	Drip loss, %	2.11 ^b^	2.62 ^a^	2.53 ^a^	2.11 ^c^	2.35 ^b^	2.46 ^b^	2.25 ^b^	1.85 ^c^	2.32 ^b^	2.00 ^bc^	2.81 ^a^	2.38 ^b^	2.38 ^b^	2.92 ^a^	0.07	<0.01	<0.01	0.01
	Cooking loss, %	62.98	61.82	63.08	62.18	61.48	62.87	63.98	62.54	62.84	62.57	62.17	61.83	60.12	63.17	0.41	0.17	0.52	0.54
	Shear force, N	39.67	37.97	39.26	37.19	39.23	39.62	38.92	38.02	42.10	39.66	39.6	36.35	36.35	39.57	0.66	0.21	0.26	0.73
Fattening Stage 2	pH_24h_	5.90	5.79	5.71	5.99	5.98	5.70	5.98	6.04	6.03	5.57	5.44	5.94	5.94	5.83	0.07	0.37	0.22	0.23
	L*	49.78 ^a^	47.35 ^b^	50.07 ^a^	46.21 ^b^	49.81 ^a^	48.18 ^ab^	50.26 ^b^	44.98 ^c^	53.72 ^a^	50.15 ^b^	49.88 ^b^	47.43 ^bc^	45.91 ^c^	46.2 ^c^	0.67	<0.01	0.01	<0.01
	a*	10.88	10.86	11.43	10.55	10.40	11.10	11.69 ^a^	10.77 ^ab^	8.93 ^b^	12.13 ^a^	11.10 ^a^	10.32 ^ab^	11.80 ^a^	10.0 ^ab^	0.26	0.97	0.34	0.01
	b*	18.11	18.22	18.25 ^ab^	16.93 ^b^	18.19 ^ab^	19.31 ^a^	18.05 ^abc^	16.00 ^c^	19.54 ^a^	18.87 ^ab^	18.44 ^ab^	17.85 ^abc^	16.85 ^bc^	19.75 ^a^	0.32	0.82	0.02	0.033
	Drip loss, %	1.98	2.19	2.35 ^a^	1.80 ^c^	2.14 ^b^	2.05 ^b^	2.34 ^a^	1.77 ^c^	1.85 ^bc^	1.98 ^bc^	2.37 ^a^	1.82 ^bc^	2.43 ^a^	2.13 ^ab^	0.05	0.10	<0.01	<0.01
	Cooking loss, %	62.38	63.52	63.75	62.86	62.89	62.29	63.99	62.16	62.51	60.85	63.52	63.56	63.26	63.74	0.34	0.11	0.522	0.39
	Shear force, N	47.57 ^a^	44.43 ^b^	47.41 ^a^	43.60 ^c^	46.46 ^bc^	46.52 ^b^	48.50 ^ab^	44.67 ^abc^	49.33 ^a^	47.78 ^ab^	46.32 ^abc^	42.53 ^c^	43.60 ^bc^	45.27 ^abc^	0.62	<0.01	<0.01	0.63

S = Songliao black; D = Duroc × Landrac × Yorkshire; A = diet with 2.92% crude fiber level; B = diet with 4.82% crude fiber level; C = diet with 6.86% crude fiber level; D = diet with 9.01% crude fiber level; G = genetic basis (breed); F = crude fiber level; G × F = interaction effect between crude fiber level and breed; L* = lightness; a* = redness; b* = yellowness. Means within the same row for the individual effects of crude fiber level, genetic basis (breed), and dietary treatments with different superscript letters differ (*p* < 0.05).

**Table 10:** Effects of dietary fiber level and breed on nutrient composition of Longissimus thoracis in finishing pigs. (%, fresh sample-basis).

Fattening Stage	Items	Breed		Crude Fiber Level (F)				Treatment								SEM	*p* Value		
		S (SLB)	D (DLY)	A (2.92%)	B (4.82%)	C (6.86%)	D (9.01%)	SA	SB	SC	SD	DA	DB	DC	DD		G	F	G × F
Fattening Stage 1	CP	25.26 ^a^	24.62 ^b^	25.05	25.13	24.78	24.80	25.47	25.57	24.94	25.05	24.63	24.69	24.61	24.56	0.90	0.03	0.59	0.75
	EE	3.79 ^a^	3.40 ^b^	3.72 ^b^	4.12 ^a^	3.37 ^c^	3.17 ^c^	3.92 ^b^	4.56 ^a^	3.49 ^cd^	3.19 ^d^	3.45 ^cd^	3.66 ^bc^	3.25 ^d^	3.15 ^d^	0.53	<0.01	<0.01	0.02
	Ash	1.81	1.87	1.57^b^	1.74 ^ab^	2.01 ^a^	2.04 ^a^	1.78 ^ab^	1.66 ^ab^	1.82 ^ab^	1.98 ^a^	1.62 ^b^	1.82 ^ab^	2.20 ^a^	2.06 ^a^	0.41	0.60	0.02	0.11
	DM	29.64	29.35	29.45	29.59	29.64	29.31	29.49	30.15	29.57	29.34	29.40	29.03	29.71	29.28	0.25	0.63	0.98	0.87
Fattening Stage 2	CP	25.90	25.64	25.75 ^ab^	26.21 ^a^	25.88 ^a^	25.26 ^b^	25.91	25.92	26.12	25.66	25.58	26.49	25.64	24.86	0.78	0.20	0.02	0.10
	EE	4.71	4.49	4.72 ^a^	4.79 ^a^	4.52 ^ab^	4.38 ^b^	4.83 ^a^	4.87 ^a^	4.60 ^ab^	4.56 ^ab^	4.61 ^ab^	4.70 ^ab^	4.43 ^ab^	4.21 ^b^	0.41	0.07	0.09	0.95
	Ash	1.41	1.33	1.34 ^ab^	1.48 ^a^	1.42 ^ab^	1.25 ^b^	1.34 ^abc^	1.41 ^ab^	1.54 ^a^	1.36 ^abc^	1.33 ^bc^	1.56 ^a^	1.29 ^abc^	1.15 ^c^	0.19	0.15	0.04	0.05
	DM	31.32	30.73	31.03	30.71	30.89	31.46	32.03	30.66	31.39	31.18	30.03	30.76	30.39	31.74	0.26	0.28	0.78	0.34

S = Songliao black; D = Duroc × Landrac × Yorkshire; A = diet with 2.92% crude fiber level; B = diet with 4.82% crude fiber level; C = diet with 6.86% crude fiber level; D = diet with 9.01% crude fiber level; G = genetic basis (breed); F = crude fiber level; G × F = interaction effect between crude fiber level and breed; CP = crude protein; EE = ether extract; DM = dry matter. Means within the same row for the individual effects of crude fiber level, genetic basis (breed), and dietary treatments with different superscript letters differ (*p* < 0.05).

**Table 11:** Effects of dietary fiber level and breed on fatty acid composition of Longissimus thoracis in finishing pigs. (%, DM-basis).

Fattening Stage	Items	Breed		Crude Fiber Level (F)				Treatment								SEM	*p* Value		
		S (SLB)	D (DLY)	A (2.92%)	B (4.82%)	C (6.86%)	D (9.01%)	SA	SB	SC	SD	DA	DB	DC	DD		G	F	G × F
Fattening stage 1	C12: 0	0.27	0.25	0.26	0.28	0.29	0.23	0.27	0.31	0.30	0.22	0.25	0.24	0.27	0.23	0.01	0.15	0.07	0.39
	C14: 0	3.93 ^a^	3.40 ^b^	3.71 ^ab^	3.54 ^b^	4.16 ^a^	3.25 ^b^	3.89	4.21	4.36	3.26	3.53	2.86	3.96	3.24	0.11	<0.01	<0.01	<0.01
	C15: 0	0.07 ^b^	0.21 ^a^	0.11 ^ab^	0.08 ^b^	0.14 ^ab^	0.22 ^a^	0.07	0.06	0.07	0.09	0.16	0.10	0.21	0.35	0.02	<0.01	<0.01	<0.01
	C16: 0	25.08 ^b^	26.41 ^a^	26.99	25.90	25.34	24.76	27.20	22.64	24.99	25.49	26.78	29.16	25.69	24.03	0.45	0.03	0.06	<0.01
	C16: 1	1.03	1.59	2.48 ^a^	0.47 ^b^	0.59 ^b^	1.70 ^a^	1.37	0.32	0.39	2.04	3.59	0.62	0.80	1.37	0.21	<0.01	<0.01	<0.01
	C17: 0	0.34 ^a^	0.29 ^b^	0.31 ^ab^	0.32 ^ab^	0.35 ^a^	0.29 ^b^	0.32	0.40	0.36	0.29	0.29	0.24	0.34	0.29	0.01	<0.01	<0.01	<0.01
	C18: 0	8.71	8.03	7.24 ^b^	9.15 ^a^	8.70 ^ab^	8.41 ^ab^	7.19	11.16	8.47	8.03	7.29	7.13	8.92	8.78	0.28	0.01	<0.01	<0.01
	C18: 1n9	43.82 ^a^	42.54 ^b^	41.39 ^b^	43.46 ^a^	43.74 ^a^	44.15 ^a^	42.67	43.13	44.63	44.88	40.11	43.78	42.86	43.43	0.30	<0.01	<0.01	<0.01
	C18: 2n6	3.40	3.34	4.12 ^a^	2.44 ^c^	3.24 ^bc^	3.69 ^ab^	3.83	2.70	4.01	3.07	4.41	2.18	2.46	4.31	0.18	0.71	<0.01	<0.01
	C18: 3n3	1.53	1.40	1.25 ^b^	1.93 ^a^	1.37 ^b^	1.31 ^b^	1.62	1.07	1.68	1.76	0.88	2.79	1.07	0.85	0.13	0.09	<0.01	<0.01
	C20: 0	0.54 ^a^	0.34 ^b^	0.73 ^a^	0.38 ^b^	0.28 ^b^	0.38 ^b^	1.18	0.41	0.24	0.32	0.27	0.34	0.32	0.43	0.06	<0.01	<0.01	<0.01
	C20: 1	2.37 ^a^	1.95 ^b^	1.97	2.31	2.29	2.07	2.29	2.99	2.37	1.82	1.64	1.64	2.21	2.31	0.12	0.03	0.47	0.02
	C20: 2	1.38 ^a^	1.20 ^b^	1.14 ^b^	1.50 ^a^	1.45 ^a^	1.08 ^b^	1.15	1.84	1.51	1.00	1.13	1.15	1.38	1.16	0.02	<0.01	<0.01	<0.01
	C22: 0	0.90	0.89	0.75 ^b^	1.12 ^a^	0.78 ^b^	0.92 ^ab^	0.59	1.63	0.50	0.86	0.90	0.61	1.06	0.99	0.08	0.92	<0.01	<0.01
	C20: 3n3	5.93 ^b^	7.00 ^a^	6.65	6.22	6.46	6.53	5.76	6.19	5.74	6.04	7.53	6.26	7.19	7.02	0.14	<0.01	0.18	<0.01
	C24: 1	0.70	1.15	0.93 ^ab^	0.92 ^b^	0.83 ^b^	1.03 ^a^	0.61	0.95	0.39	0.84	1.25	0.89	1.26	1.21	0.06	<0.01	<0.01	<0.01
	SFA	39.84	39.82	40.08 ^a^	40.76 ^a^	40.03 ^a^	38.45 ^b^	40.70	40.81	39.28	38.56	39.47	40.70	40.78	38.34	0.28	0.98	0.02	0.23
	MUFA	47.92	47.24	46.76 ^b^	47.16 ^b^	47.45 ^b^	48.95 ^a^	46.94	47.39	47.78	49.57	46.59	46.92	47.12	48.32	0.22	0.98	0.02	0.23
	PUFA	12.25	12.94	13.15 ^a^	12.09 ^b^	12.52 ^ab^	12.60 ^ab^	12.36	11.80	12.94	11.87	13.94	12.37	12.10	13.34	0.18	0.06	0.04	<0.01
Fattening stage 2	C12: 0	0.31 ^a^	0.28 ^b^	0.27 ^b^	0.25 ^b^	0.32 ^a^	0.34 ^a^	0.27	0.30	0.36	0.31	0.27	0.21	0.29	0.37	0.01	0.02	<0.01	<0.01
	C14: 0	4.31 ^a^	3.89 ^b^	3.93 ^bc^	3.45 ^c^	4.44 ^ab^	4.60 ^a^	3.89	3.93	4.88	4.55	3.96	2.97	3.99	4.65	0.13	<0.01	<0.01	<0.01
	C15: 0	0.11 ^a^	0.06 ^b^	0.08 ^ab^	0.11 ^a^	0.08 ^ab^	0.06 ^b^	0.11	0.16	0.09	0.07	0.06	0.06	0.06	0.06	0.01	<0.01	<0.01	<0.01
	C16: 0	21.89 ^b^	23.53 ^a^	26.83 ^a^	24.16 ^ab^	21.03 ^bc^	18.82 ^c^	26.85	20.65	19.85	20.21	26.82	27.67	22.22	17.42	0.81	0.03	<0.01	<0.01
	C16: 1	0.59	0.52	0.58 ^ab^	0.58 ^ab^	0.63 ^a^	0.43 ^b^	0.48	0.75	0.71	0.40	0.68	0.41	0.56	0.46	0.03	0.11	<0.01	<0.01
	C17: 0	0.38 ^a^	0.32 ^b^	0.33 ^b^	0.31 ^b^	0.37 ^ab^	0.42 ^a^	0.35	0.39	0.41	0.38	0.30	0.23	0.32	0.45	0.01	<0.01	<0.01	<0.01
	C18: 0	9.84 ^a^	8.03 ^b^	8.54	8.13	9.91	9.16	8.67	8.87	10.90	10.94	8.41	7.40	8.91	7.38	0.33	0.01	0.09	0.15
	C18: 1n9	40.56 ^b^	45.47 ^a^	41.78 ^b^	39.99 ^b^	43.86 ^ab^	46.43 ^a^	40.08	35.85	42.01	44.29	43.47	44.12	45.72	48.57	0.78	<0.01	<0.01	0.02
	C18: 2n6	5.83 ^a^	4.31 ^b^	4.05 ^b^	5.81 ^a^	5.05 ^ab^	5.38 ^ab^	4.08	7.83	5.74	5.67	4.02	3.79	4.35	5.10	0.27	<0.01	<0.01	<0.01
	C18: 3n3	0.74	0.59	0.56	0.78	0.64	0.69	0.72	0.89	0.66	0.68	0.39	0.67	0.61	0.69	0.04	0.08	0.28	0.43
	C20: 0	2.67 ^a^	0.41 ^b^	1.69 ^ab^	3.65 ^a^	0.42 ^b^	0.41 ^b^	2.99	6.91	0.39	0.38	0.40	0.38	0.45	0.42	0.46	<0.01	<0.01	<0.01
	C20: 1	2.76 ^b^	3.29 ^a^	2.49 ^b^	2.32 ^b^	3.37 ^a^	3.93 ^a^	2.12	2.17	3.55	3.23	2.86	2.46	3.19	4.64	0.17	<0.01	<0.01	<0.01
	C20: 2	1.38 ^b^	1.54 ^a^	1.23 ^b^	1.34 ^b^	1.51 ^ab^	1.74 ^a^	1.25	1.39	1.58	1.28	1.21	1.28	1.46	2.20	0.07	0.02	<0.01	<0.01
	C22: 0	0.70 ^a^	0.24 ^b^	0.56	0.57	0.42	0.33	0.65	0.93	0.71	0.51	0.46	0.22	0.14	0.15	0.06	<0.01	<0.01	<0.01
	C20: 3n3	7.19 ^a^	6.74 ^b^	6.43 ^c^	7.78 ^a^	7.13 ^b^	6.52 ^c^	6.79	8.27	7.33	6.36	6.06	7.29	6.93	6.69	0.14	<0.01	<0.01	<0.01
	C24: 1	0.70	0.77	0.68 ^b^	0.79 ^ab^	0.83 ^a^	0.66 ^b^	0.70	0.72	0.83	0.55	0.64	0.85	0.83	0.76	0.03	0.07	0.01	0.07
	SFA	40.21 ^a^	36.77 ^b^	42.22 ^a^	40.63 ^a^	36.98 ^b^	34.12 ^b^	43.77	42.13	37.60	37.35	40.67	39.13	36.37	30.90	0.83	<0.01	<0.01	0.12
	MUFA	44.61 ^b^	50.05 ^a^	45.52 ^bc^	43.67 ^c^	48.69 ^ab^	51.45 ^a^	43.38	39.50	47.09	48.47	47.65	47.84	50.29	54.43	0.89	<0.01	<0.01	0.10
	PUFA	15.13 ^a^	13.18 ^b^	12.26 ^b^	15.70 ^a^	14.33 ^ab^	14.33 ^ab^	12.84	18.38	15.31	13.99	11.68	13.03	13.35	14.67	0.41	<0.01	<0.01	0.02

S = Songliao black; D = Duroc × Landrac × Yorkshire; A = diet with 2.92% crude fiber level; B = diet with 4.82% crude fiber level; C = diet with 6.86% crude fiber level; D = diet with 9.01% crude fiber level; G = genetic basis (breed); F = crude fiber level; G × F = interaction effect between crude fiber level and breed; SFA = saturated fatty acid; MUFA = monounsaturated fatty acids; PUFA = polyunsaturated fatty acids. Means within the same row for the individual effects of crude fiber level, genetic basis (breed), and dietary treatments with different superscript letters differ (*p* < 0.05).

## Data Availability

The original contributions presented in the study are included in the article; further inquiries can be directed to the corresponding authors.
